# Impact of Age and Menopausal Status on T-DM1 (Ado-Trastuzumab Emtansine) Treatment Outcomes in HER2-Positive Breast Cancer

**DOI:** 10.3390/ph18060931

**Published:** 2025-06-19

**Authors:** Heves Surmeli, Deniz Isik, Oguzcan Kinikoglu, Yunus Emre Altintas, Ugur Ozkerim, Sıla Oksuz, Tugba Basoglu, Hatice Odabas, Nedim Turan

**Affiliations:** Department of Medical Oncology, Kartal Dr. Lütfi Kirdar City Hospital, Health Science University, İstanbul 34865, Türkiye; dnz.1984@yahoo.com (D.I.); ogokinikoglu@yahoo.com (O.K.); yunusaltintas1688@gmail.com (Y.E.A.); ugur.ozkerim@hotmail.com (U.O.); sila.oksuz@gmail.com (S.O.); basoglutugba@gmail.com (T.B.); odabashatice@yahoo.com (H.O.); turan.nedim@hotmail.com (N.T.)

**Keywords:** breast cancer, menopausal status, Trastuzumab Deruxtecan (TDM-1), treatment response, side effects, metastasis, survival

## Abstract

**Background/Objectives**: HER2-positive breast cancer is an aggressive subtype with an established responsiveness to HER2-targeted therapies like ado-trastuzumab emtansine (T-DM1). However, inter-patient variability in treatment response and toxicity remains a challenge. Hormonal status, particularly menopausal state, may influence breast cancer behavior, therapeutic tolerance, and outcomes, yet data on its effect in patients treated with T-DM1 are scarce. This study aimed to evaluate whether menopausal status independently affects treatment response, side effects, and survival outcomes in HER2-positive breast cancer patients receiving T-DM1, accounting for the confounding role of age. **Methods**: This retrospective cohort study included 98 female patients with HER2-positive breast cancer treated with T-DM1: 53 premenopausal and 45 postmenopausal. The clinical characteristics, metastatic patterns, treatment history, T-DM1 outcomes, and toxicities were recorded. The statistical analysis included chi-square, *t*-tests, Mann–Whitney U tests, and Spearman’s correlations. Partial correlation analyses were conducted to isolate the effect of menopausal status by controlling for age. **Results**: The postmenopausal patients showed higher rates of lung metastasis (42.2% vs. 20.8%) and mortality (60.0% vs. 39.6%) than premenopausal patients. However, no significant differences were found in the T-DM1 response or toxicity profiles. After adjusting for age, menopausal status had no independent association with the treatment outcomes or side effects. Age was the dominant factor influencing performance status, metastatic burden, and mortality risk. **Conclusions**: Menopausal status affects disease presentation but not T-DM1 efficacy or toxicity when age is accounted for. Treatment decisions should consider age and clinical profile rather than menopausal classification alone when managing HER2-positive breast cancer with T-DM1.

## 1. Introduction

### 1.1. Background

Breast cancer is the most commonly diagnosed malignancy among women worldwide and a leading cause of cancer-related mortality [1]. HER2-positive breast cancer accounts for approximately 15–20% of all cases and is associated with aggressive behavior and a poor prognosis [2,3]. However, the introduction of HER2-targeted therapies, including trastuzumab emtansine (T-DM1), has significantly improved clinical outcomes [4]. T-DM1 is an antibody–drug conjugate combining trastuzumab with a cytotoxic agent and is approved for HER2-positive metastatic or unresectable breast cancer based on studies such as the EMILIA and TH3RESA trials [4,5].

### 1.2. Hormonal Influence and Menopausal Status

Hormonal dynamics, particularly estrogen and progesterone levels, play a critical role in breast cancer biology. Menopausal status influences circulating hormone levels and may indirectly affect tumor behavior and treatment response [6]. Premenopausal women tend to have more aggressive tumors and higher estrogen levels, while postmenopausal women typically present with less proliferative tumors but may experience more comorbidities and age-related physiological decline [7].

### 1.3. Clinical Relevance of Menopausal Status

Menopausal status is associated with differences in treatment tolerance, metastatic patterns, and prognosis. Postmenopausal women often have a higher risk of visceral metastasis, such as in the lung and liver, while premenopausal patients are more likely to experience bone involvement. In addition, age-related organ function decline may influence drug metabolism and toxicity profiles, especially with cytotoxic agents like T-DM1 [7].

### 1.4. Rationale and Research Gap

Despite these known differences, pivotal clinical trials on T-DM1 have not stratified patients by menopausal status [5,8]. Consequently, it remains unclear whether T-DM1’s efficacy and safety differ based on menopausal state. The existing literature has suggested possible hormonal modulation of HER2 signaling, but the findings specific to T-DM1 remain limited [9].

### 1.5. Objective

This study aims to evaluate whether menopausal status independently influences treatment response and side effects of T-DM1 in HER2-positive breast cancer patients, considering the confounding role of age.

## 2. Results

### 2.1. Patient and Disease Characteristics by Menopausal Status (Table 1)

The table above compares the characteristics of the premenopausal (n = 53) and postmenopausal (n = 45) women with breast cancer. We found that the premenopausal women were younger on average (49.07 vs. 69 years) and diagnosed earlier (39.81 vs. 59.07 years), *p* values < 0.01, meaning that there were significant differences. Higher premenopausal women’s ECOG normal (fully active) indicated better physical function (96.2% vs. 66.7%, *p* < 0.01). No difference was found (*p* = 0.235) in invasive ductal carcinoma rates (60.4% vs. 53.3%). No other notable differences in stage, grade, or biomarkers were observed (*p* > 0.05). The *p*-values whether low (<0.01) or high (>0.05), statistically significant or not, as to whether there exists or is evidence for a difference. In brief, there are significant differences between age and physical function and large between-group differences in the cancer type, stage, and biomarkers.

**Table 1:** Patient and disease characteristics by menopausal status.

Characteristic	Premenopausal (n = 53)	Postmenopausal (n = 45)	*p*-Value
**Age (years)**	49.07 ± 7.98	69 ± 8.38	<0.01
**Diagnosis Age (years)**	39.81 ± 6.24	59.07 ± 8.01	<0.01
**ECOG Normal (%)**	96.2	66.7	<0.01
**Invasive Ductal (%)**	60.4	53.3	0.235
**Stage, Grade, Biomarkers**	No significant difference	No significant difference	>0.05

**Figure 1:**
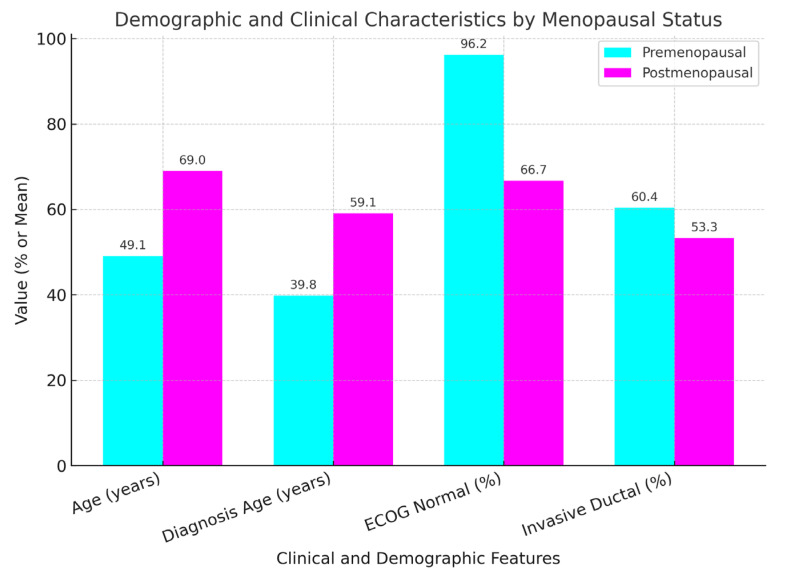
Comparison of age, diagnosis age, and ECOG performance status.

The premenopausal patients were significantly younger at diagnosis and exhibited higher rates of normal ECOG performance status compared with postmenopausal patients (*p* < 0.01) in Figure 1.

### 2.2. Metastatic Regions by Menopausal Status

**Figure 2:**
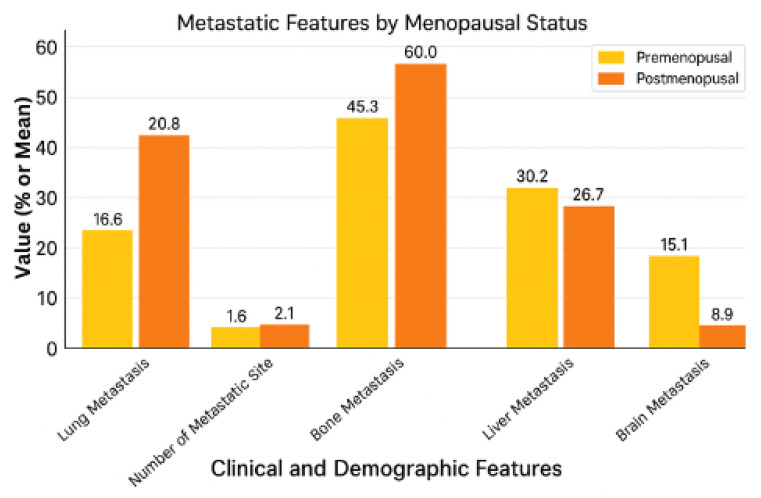
Distribution of metastatic sites by menopausal status.

Table 2 presents the statistical comparisons of metastatic sites between the groups, while Figure 2 offers a visual summary of these differences for easier interpretation of distribution patterns by menopausal status. Table 2 compares metastatic regions in breast cancer patients by menopausal status. The difference between the postmenopausal and premenopausal patients in terms of frequency of lung metastasis is statistically significant (*p* = 0.038). Regarding metastatic sites, there is also a big difference between the postmenopausal and premenopausal patients, as there are 2.07 ± 1.21 sites (mean ± SD) against postmenopausal patients and 1.58 ± 1.05 in premenopausal patients (*p* = 0.030), which indicates greater metastatic spread post menopause. No other metastatic sites, including bone (45.3 vs. 60.0%, *p* = 0.211), liver (30.2 vs. 26.7%, *p* = 0.873), and brain (15.1 vs. 8.9%, *p* = 0.532), differed significantly between the groups (*p* > 0.05). In other words, these findings indicate that postmenopausal patients are more likely to develop lung metastasis and a more significant number of metastatic sites, which is potentially indicative of differing aggressiveness or hormonal influences in the disease. Nevertheless, while having no significance in other areas, these patterns of metastases do not uniformly indicate the effect of menopausal status, suggesting that further study into underlying biological factors is warranted [10].

A bar graph illustrating the frequency of lung, bone, liver, and brain metastases, along with the mean number of metastatic sites is shown in Figure 2. Lung metastases and total metastatic burden were significantly higher in the postmenopausal patients.

### 2.3. Treatment History by Menopausal Status

Table 3 compares the treatment history by menopausal status. Treatment at an early stage has no significant difference in de novo metastasis (32.1% vs. 33.3%, *p* = 1.000), neoadjuvant (39.6% vs. 33.3%, *p* = 0.665), and adjuvant therapies (64.2 vs. 64.4%, *p* = 1.000). Additionally, the significance of adjuvant hormonal treatment (50.9% vs. 33.3%, *p* = 0.121) and radiotherapy (67.9% vs. 64.4%, *p* = 0.882) are insignificant. HER2 therapy rates are similar to pre-TDM1 treatment (79.2% vs. 80.0%, *p* = 1.000) and there was no statistically significant difference in hormonal therapy (47.2% vs. 35.6%, *p* = 0.339). This suggests that the treatment pattern is not markedly affected by the menopausal status, though some variations might reflect clinical preference.

**Figure 3:**
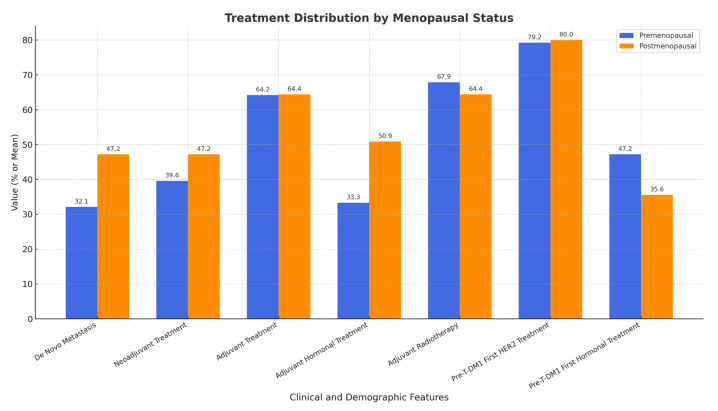
Treatment history prior to T-DM1.

A comparison of early-stage treatment interventions and prior HER2/hormonal therapies between groups is shown in Figure 3. No statistically significant differences were observed across the treatment categories.

### 2.4. TDM-1 Treatment Response and Side Effects

In Table 4, the effect of TDM1 treatment on menopausal status and its side effects is evaluated. However, in terms of the brain (28.3% vs. 31.1%, *p* = 0.935), liver (13.2% vs. 28.9%, *p* = 0.095), lung (20.8% vs. 33.3%, *p* = 0.240) and bone (24.5% vs. 33.3%, *p* = 0.461), no significant difference is observed in progression during TDM-1 treatment. There are no differences in side effects such as dose delay (9.4% vs. 6.7%, *p* = 0.898), discontinuation (5.7% vs. 6.7%, *p* = 1.000), or adverse events (15.1% vs. 8.9%, *p* = 0.532). Regarding mortality, postmenopausal patients (60.0%) are closer to significance (*p* = 0.071) than age, sex, and BMI-matched patients (39.6%). However, there is no significant difference in the overall survival (6.23 vs. 5.63 years, *p* = 0.477) between menopausal status and TDM-1 outcomes.

**Figure 4:**
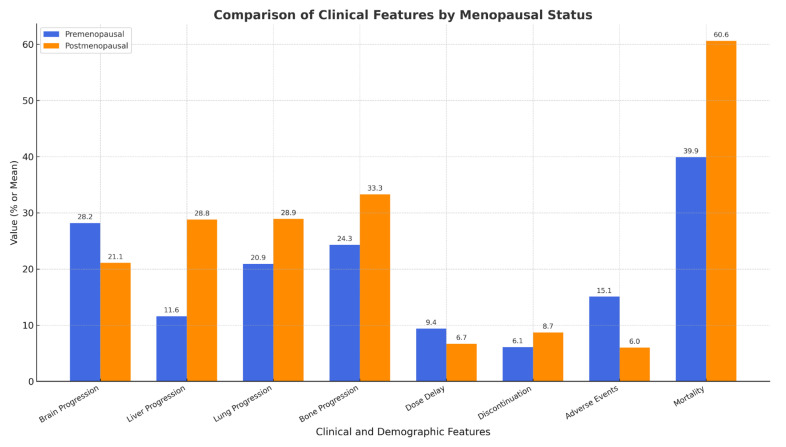
T-DM1 treatment response and adverse events.

A visual representation of organ-specific progression rates, dose delays, discontinuation rates, and adverse events is shown in Figure 4. No significant differences were observed between the premenopausal and postmenopausal patients.

### 2.5. Correlation Analysis

The correlations of several factors with menopausal status are shown in Table 5. Age (r = 0.798, *p* < 0.01), diagnosis age (r = 0.837, *p* < 0.01), and age in postmenopausal patients show significant positive correlations. This worsens the functional status of postmenopausal ECOG performance (r = 0.389, *p* < 0.01). Lung metastasis (r = 0.232, *p* < 0.05) and mortality (r = 0.203, *p* < 0.05) are also significantly correlated, with increasing risk post menopause. The TDM-1 response of brain progression, adverse events, and metastatic sites (except bone) have nonsignificant correlations with menopausal status (*p* > 0.05, r = 0.031, r = −0.094, and r = 0.147, respectively).

**Figure 5:**
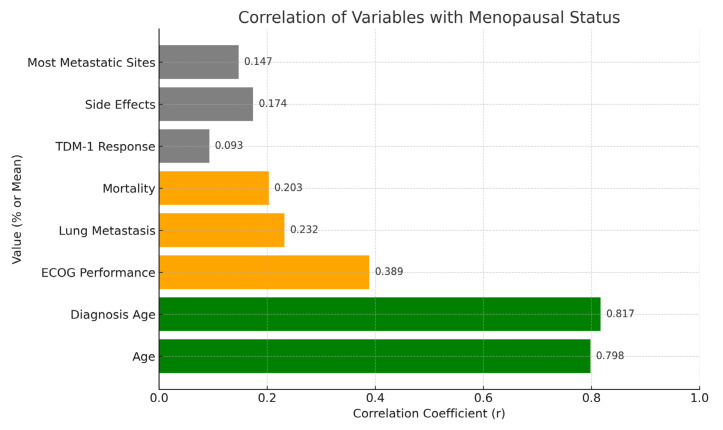
Spearman’s correlation matrix: menopausal status vs. clinical outcomes.

A correlation map displaying the strength and direction of the associations between menopausal status and various clinical parameters is shown in Figure 5. Significant positive correlations were identified for age, ECOG performance, lung metastasis, and mortality.

### 2.6. Partial Correlation Controlling for Age

Table 6 reports the partial correlations between the menopausal status and variables corrected for age. The analysis does not find any significant correlations (*p* > 0.05). There is a weak positive correlation between ECOG performance (r = 0.213), which does not reach significance and indicates slightly less function after menopause. Menopause is found to have a negligible correlation (r = 0.038) to lung metastasis and, thus, has no independent link to metastasis to the lung. There is also no significant effect of menopause beyond age in other parameters such as mortality (r = −0.137) and overall survival (r = −0.287), which each have a weak negative association that indicates nowhere near a strong menopausal effect. Our findings indicate that such age effects, as part of age, explain most of the previously described differences (e.g., performance, metastasis, and mortality) at least in part, rather than anticancer therapy alone.

**Figure 6:**
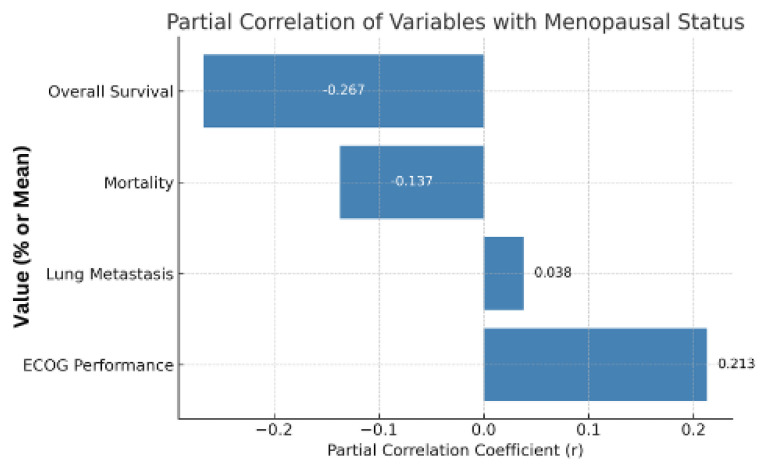
Partial correlation analysis controlling for age.

A graphical output showing partial correlation coefficients after adjusting for age is shown in Figure 6. No significant independent associations were observed between menopausal status and performance status, metastasis, mortality, or survival outcomes.

## 3. Discussion

### 3.1. Interpretation of Findings

The differences in age and ECOG performance status between the premenopausal and postmenopausal groups for patient and disease characteristics suggest age-based aging more than menopause-specific effects. Since there is no significant variation in tumor biomarkers (i.e., estrogen or progesterone receptor positivity), the underlying cancer biology could be reinforced to be similar to menopausal status with little hormonal impact on the tumor traits. In the metastatic patterns, the rates of lung metastases and the number of metastatic sites are increased in postmenopausal patients compared with younger patients, which may reflect disease progression with increased age rather than a unique danger of menopause. The patients in both groups were treated similarly for early-stage and pre-TDM1 therapies, which signifies that clinical management had a uniform approach across menopausal statuses. Regarding the TDM-1 outcomes, adjusting for age, there is no independent effect of menopausal status on treatment response or side effects, and age remains the primary characteristic. Yet, a trend towards higher mortality in postmenopausal patients (*p* = 0.071), which could not be attributed to menopause but to the higher rates of comorbidities or a more significant burden of disease in elderly patients, is, however, observed. Based on these findings, age is the main confounder that overshadows menopausal status in the observed differences.

### 3.2. Comparison with the Literature

Breast cancer outcomes in the elderly have been well described. SEER database analyses and studies show that younger patients (<40) of triple-negative or HER2-positive cancers do not fare as well as older patients when it comes to recurrence and survival. Age-specific prognostic factors match research showing that tumor biology, delayed diagnosis, and robust immune response are the contributors. As a reverse finding, older patients (≥65) may have poorer outcomes, particularly if associated with comorbidities, less intensive therapies, or reduced treatment tolerance, as is the case for geriatric oncology. The patterns of these observations underscore age as the most important variable in prognosis and treatment planning. While age is one issue, hormonal status is another and, in the latter case, targets therapy efficacy more than age. However, endocrine therapies such as tamoxifen or aromatase inhibitors are effective across aging groups in treating hormone receptor-positive (HR+) cancers, as shown by clinical trials such as ATAC and BIG 1-98. Despite this, premenopausal HR+ patients require ovarian suppression rather than age. The CRISPR-mediated delivery of kinase demonstrates efficacy in both preclinical and clinical models similar to HER2 targeted therapies (e.g., trastuzumab) that have context-specific-, not age, based treatment and show distinct effects on HR-negative and HER2-positive tumors in the neoadjuvant setting. In contrast to the traditional age-centric studies, the elements of age that are of concern tend to be framed as hormonal and receptor status, which are most predictive of therapeutic success [11].

Furthermore, comparisons with other HER2-targeted therapies, such as trastuzumab and pertuzumab, suggest that the interaction between hormonal status and HER2 inhibition may be nuanced. In a pooled analysis by [12], premenopausal patients receiving adjuvant trastuzumab demonstrated better disease-free survival, potentially linked to age, hormonal receptor status, or treatment adherence. Similarly, the CLEOPATRA study showed that pertuzumab plus trastuzumab and docetaxel improved the outcomes irrespective of menopausal status, indicating a possibly lesser role of menopausal state in determining HER2-targeted therapy response. These findings parallel our observation that menopausal status alone did not independently influence T-DM1 response after adjusting for age. Moreover, research indicates that estrogen signaling may crosstalk with HER2 pathways, potentially altering treatment dynamics; however, clinical outcomes appear to be more significantly shaped by age and comorbidities than menopausal status alone [13,14]. Such comparisons reinforce the need to disentangle age and hormonal effects in interpreting treatment efficacy in HER2-positive breast cancer.

### 3.3. Clinical Implications

TDM-1 (ado-trastuzumab emtansine) treatment should not be based solely on menopausal status. TDM 1 is a HER2-targeted antibody–drug conjugate indicated for HER2-positive metastatic breast cancer and has been proven efficacious based on trials such as EMILIA and KATHERINE. These studies show that the benefit is consistent across premenopausal and postmenopausal patients, suggesting that HER2 overexpression rather than menopausal status is responsible for the therapeutic response. Failing to consider other tumor-specific factors (e.g., receptor expression, prior disease treatment resistance) or menopausal status, which have a profound impact on outcomes, specifically in a pelvic tumor setting, means focusing only on menopausal status. For this reason, treatments need to be based mainly on molecular and clinical factors and not on hormonal classification to maximize efficacy. The effective use of TDM-1 requires individualized care, factoring in age and metastatic profile. Due to younger patients often having more aggressive diseases and fewer comorbidities than older patients, TDM-1 can be tolerated in younger patients. In contrast, older patients may run into dose-limiting toxicities due to age-related physiological decline or polypharmacy. Also, therapy is guided by metastatic patterns as the visceral involvement usually requires immediate treatment with TDM-1, regardless of age, compared with bone-only disease. This approach further integrates genomic profiling and performance status to define a more precise approach. This comprehensive framework ensures the maximum benefits of TDM-1 in heterogeneous patient populations [15].

### 3.4. Future Directions

The current limitations must be overcome, and the evidence must be strengthened by introducing prospective studies with larger cohorts. Prospective trials, however, provide the ideal design to show temporality and causality as they track patients from baseline through to treatment and outcomes. The statistical power would be increased with larger sample sizes, which would aid in detecting slight differences in TDM-1 efficacy by age, menopausal status, or metastatic profile. This would minimize type II errors, enhance precision, and provide robust data to inform clinical decision making. Furthermore, adding diversity to the patient populations would also improve generalizability, addressing a gap in understanding patient demographics and their role in determining therapeutic response. Just as important as the inclusion of molecular profiling is the identification of the underlying mechanisms that explain the observed outcome. Such genomic and proteomic analyses as next-generation sequencing or expression RNA may elucidate the biomarkers associated with TDM-1 sensitivity or resistance. For example, different responses may be related to clinical factors or variations in HER2 amplification status, PIK3CA mutations, or immune microenvironment signature. These data could be integrated with patient characteristics to explain why one subpopulation may benefit more and pave the way for precision oncology. Mechanistic insights such as these would inform targeted trial designs and treatment strategies in a manner that is consonant with the growing trend in the field to devolve into molecularly defined care and to achieve improved outcomes in HER2-positive breast cancer.

### 3.5. Strengths and Limitations

This study has several strengths. It addresses an underexplored area of clinical importance by evaluating the impact of menopausal status on T-DM1 treatment outcomes in HER2-positive breast cancer patients (Table 7). The retrospective cohort was well defined, and the key variables were analyzed with appropriate statistical methods, including partial correlation to control for the confounding effect of age. This study also provides a comprehensive view by integrating clinical features, metastatic patterns, treatment responses, and toxicity profiles.

However, this study has limitations. Its retrospective design may introduce selection bias, and the reliance on electronic medical records may limit data completeness or standardization across all patients. The relatively small sample size (n = 98) limits statistical power to detect smaller effect sizes, especially in subgroup analyses. Additionally, the absence of stratified molecular profiling, such as HER2 expression levels or PIK3CA mutations, restricts our ability to explore the mechanistic explanations for the observed patterns. Finally, although age-adjusted analyses were performed, menopausal status may still be partially confounded with unmeasured age-related variables.

## 4. Materials and Methods

### 4.1. Study Design

In this study, a retrospective cohort analysis was performed to evaluate breast cancer patients given Trastuzumab Deruxtecan (TDM-1). Female patients with HER2-positive breast cancer who received TDM-1 were included from clinical records. The 98 patients were classified by menopausal status at treatment initiation into premenopausal patients (53) and postmenopausal patients (45). This study could analyze this historical clinical, pathological, and treatment data without intervention in a prospective fashion, using records from before 7 April 2025. TDM-1 efficacy and toxicity, based on menopausal groups, were documented. Although documented information was leveraged, new patient interventions were avoided, making the study feasible from the ethical perspective and enabling the evaluation of treatment patterns, disease progression, and survival. This methodology facilitates an appropriate primary investigation into how menopausal status impacts TDM-1 performance within a large enough sample to identify subgroup differences.

### 4.2. Patient Population

This study consisted of female patients with breast cancer treated with Trastuzumab Deruxtecan (TDM-1). The breast cancer diagnosis was required to be confirmed at any stage, TDM-1 therapy was documented at a designated medical facility, and the requirement for inclusion criteria included confirmation of breast cancer diagnosis. Patients were classified as premenopausal or postmenopausal according to menopausal status at treatment start. Limitations were also set on exclusion criteria, including incompleteness of medical records or absence of menopausal status data so that group classification was reliable and outcome assessment was comprehensive. In addition, the records that lacked clinical, pathological, and treatment details were excluded to maintain the data integrity. The cohort available for retrospective analysis had 53 premenopausal and 45 postmenopausal individuals, constituting 98 patients in the total sample size. The effect size was set with G*Power 3.1.9.4 software to d = 0.8 as a large effect size, alpha of 0.05, and power of 97.4%. This calculation guaranteed that statistical robustness in observing significant differences in TDM-1 efficacy and toxicity between menopausal groups required a minimum of 98 patients. This resulting cohort size is amenable to evaluating menopausal status’s effect on treatment outcomes in a well-defined population that can generate reliable conclusions. Only patients with invasive ductal carcinoma were included to maintain histological homogeneity and reduce variability in treatment response associated with different breast cancer subtypes. This approach allowed for a more focused analysis of menopausal status without introducing additional confounding from rarer histological types.

### 4.3. Data Collection

Clinical and demographic data were collected retrospectively from patient records, including age, ECOG performance status, tumor biomarkers (ER, PR, HER2, and Ki67), and metastatic sites. Treatment details—such as prior therapies, T-DM1 response, toxicity, and survival outcomes—were documented. Only complete records with verified menopausal status were included, totaling 98 patients (53 premenopausal, 45 postmenopausal).

### 4.4. Statistical Analysis

Continuous variables were summarized using means and standard deviations; categorical variables using frequencies and percentages. Between-group comparisons used the Independent Samples T-test or Mann–Whitney U test, as appropriate. Categorical variables were analyzed with chi-square tests. Correlation analysis (Spearman’s) assessed associations between menopausal status and key outcomes. Partial correlations were used to control for age and isolate its confounding effect. A *p*-value < 0.05 was considered statistically significant. Analyses were performed using SPSS 26.0.

## 5. Conclusions

### 5.1. Summary

Menopausal status has an impact on breast cancer dynamics (e.g., age at diagnosis, performance status, prevalence of lung metastasis, and risk of death). Still, it does not independently affect the outcome of TDM-1 (ado-trastuzumab emtansine) treatment beyond age-related effects. Younger premenopausal patients tend to have robust performance but aggressive disease biology, including high rates of lung metastasis and higher death. Older patients (postmenopausal) have reduced performance from age-related decline and show specific metastatic patterns and survival risk from comorbidities. Nevertheless, HER2 status and not menopausal status was found to be the key determinant of TDM-1 efficacy in this study on HER2-positive metastatic breast cancer. These relationships are modulated by age, affecting the tolerance of treatment and the progression of disease; but, menopausal status does not have a corresponding independent impact on TDM-1 response. For instance, this implies that although menopausal status influences clinical presentation and outcome, hormonal categorization should not be the most predominant factor in therapeutic decisions for TDM-1; rather, age, metastatic profile, and molecular characteristics should be considered individually.

### 5.2. Significance

The findings of this study stress the importance of age as a confounding factor in the analysis of menopausal status effects on breast cancer outcomes. Despite their association with disease presentation, such as lung metastasis and mortality, TDM-1 treatment response is not dependent on menopausal status alone but is independent of age. Performance status and treatment tolerance, as well as metastatic behavior, are dependent on age and can correlate with menopausal transitions; young premenopausal patients have more aggressive disease, and postmenopausal patients develop comorbidities that modify prognosis. These findings challenge the simplistic use of menopausal status for therapeutic decisions, in particular in the setting of HER2-positive cases, given the unexpected findings of association by use of age as a confounder. However, this distinction is crucial since it establishes an age-related physiological and tumor-specific focus for clinical questioning instead of relying on equality as a surrogate for the hormonal state. It calls for the disentangling of these variables for research and practice so that age effects are considered in treatment strategies. This insight further narrows our understanding of patient heterogeneity beyond broader categorizations, like menopause, where precision in oncology is called for in the clinic.

### 5.3. Recommendations

Because treatment of HER2-positive breast cancer, particularly in conjunction with TDM-1, should rest on the entire patient profile and not just menopausal status, future research and clinical trial design will increasingly take into account such patient characterizations. Age alone accounts for the variation in this study’s status variables, such as age, performance, and metastasis. When age is accounted for, menopausal status does not independently predict the outcome of TDM-1 treatment. For this reason, clinical assessments should include age, metastatic pattern, performance status, and a molecular characterization such as the HER2 expression levels or genomic alterations. Aggressive management of visceral disease is appropriate for younger patients, with adjustments for comorbidities and frailty needed for older patients. Further precision can be achieved by incorporating genomic profiling to refine therapy further, by identifying resistance markers or predictive biomarkers. This shift helps to de-emphasize menopausal status as the sole criterion for the consideration of treatment, consistent with other data that indicate that age and tumor biology, rather than menopausal status, are determinants of response to therapy. Therefore, individualized strategies should be adopted, such as TDM-1 enhanced therapeutic efficacy, low overtreatment or under treatment, and the implementation of patient-centered oncology care.

## Figures and Tables

**Table 2:** Metastatic regions by menopausal status.

Metastatic Feature	Premenopausal (n = 53)	Postmenopausal (n = 45)	*p*-Value
**Lung Metastasis**	20.8% (11/53)	42.2% (19/45)	0.038 *
**Number of Metastatic Sites**	1.58 ± 1.05 (Mean ± SD)	2.07 ± 1.21 (Mean ± SD)	0.030 *
**Bone Metastasis**	45.3% (24/53)	60.0% (27/45)	0.211
**Liver Metastasis**	30.2% (16/53)	26.7% (12/45)	0.873
**Brain Metastasis**	15.1% (8/53)	8.9% (4/45)	0.532

(* *p* < 0.05 indicates statistical significance).

**Table 3:** Treatment history by menopausal status.

Treatment Feature	Premenopausal (n = 53)	Postmenopausal (n = 45)	*p*-Value
**De Novo Metastasis**	32.1% (17/53)	47.2% (25/53)	1.000
**Neoadjuvant Treatment**	39.6% (21/53)	47.2% (25/53)	0.665
**Adjuvant Treatment**	64.2% (34/53)	64.4% (29/45)	1.000
**Adjuvant Hormonal Treatment**	50.9% (27/53)	33.3% (15/45)	0.121
**Adjuvant Radiotherapy**	67.9% (36/53)	64.4% (29/45)	0.882
**Pre-TDM-1 First HER2 Treatment**	79.2% (42/53)	80.0% (36/45)	1.000
**Pre-TDM-1 First Hormonal Treatment**	47.2% (25/53)	35.6% (16/45)	0.339

**Table 4:** Treatment response and side effects.

Feature	Premenopausal (n = 53)	Postmenopausal (n = 45)	*p*-Value
Brain Progression	28.3% (15/53)	31.1% (14/45)	0.935
Liver Progression	13.2% (7/53)	28.9% (13/45)	0.095
Lung Progression	20.8% (11/53)	33.3% (15/45)	0.240
Bone Progression	24.5% (13/53)	33.3% (15/45)	0.461
Dose Delay	9.4% (5/53)	6.7% (3/45)	0.898
Discontinuation	5.7% (3/53)	6.7% (3/45)	1.000
Adverse Events	15.1% (8/53)	8.9% (4/45)	0.532
Mortality	39.6% (21/53)	60.0% (27/45)	0.071
Overall Survival (years)	6.23 ± 4.23	5.63 ± 4.13	0.477

**Table 5:** Correlation analysis.

Variable	Correlation with Menopause (r)	*p*-Value
Age	0.798	<0.01
Diagnosis Age	0.837	<0.01
ECOG Performance	0.389	<0.01
Lung Metastasis	0.232	<0.05
Mortality	0.203	<0.05
TDM-1 Response (e.g., Brain Progression)	0.031	0.935
Side Effects (e.g., Adverse Events)	−0.094	0.532
Most Metastatic Sites (e.g., Bone)	0.147	0.147

**Table 6:** Partial correlation controlling for age.

Variable	Partial Correlation with Menopause (r)	*p*-Value
ECOG Performance	0.213	>0.05
Lung Metastasis	0.038	>0.05
Mortality	−0.137	>0.05
Overall Survival	−0.287	>0.05

**Table 7:** Summary of key findings comparing premenopausal and postmenopausal breast cancer patients treated with T-DM1.

Feature	Premenopausal	Postmenopausal	*p*-Value
Age (mean ± SD)	49.07 ± 7.98	69.00 ± 8.38	<0.01
ECOG Normal (%)	96.2%	66.7%	<0.01
Lung Metastasis (%)	20.8%	42.2%	0.038
Number of Metastatic Sites (mean)	1.58	0.07	0.030
Mortality (%)	39.6%	60.0%	0.071
T-DM1 Response/Side Effects	No significant difference	No significant difference	>0.05
Correlation with Menopause (Mortality)	r = 0.203		<0.05
Independent Effect After Controlling for Age	Not significant		>0.05

## Data Availability

The original contributions presented in this study are included in the article. Further inquiries can be directed to the corresponding author.
